# Supplementary material for: GWAS of serum ALT and AST reveals an association of SLC30A10 Thr95Ile with hypermanganesemia symptoms
Source: Nat Commun. 2021 Jul 27;12:4571. doi: 10.1038/s41467-021-24563-1 (PMC8316433; doi:10.1038/s41467-021-24563-1)
Supplement: Supplementary file 1 — Supplementary Information [file 41467_2021_24563_MOESM1_ESM.pdf]

Supplementary Information for

GWAS of serum ALT and AST reveals an association of *SLC30A10* Thr95Ile with hypermanganesemia symptoms

Lucas D. Ward[1*], Ho-Chou Tu[1], Chelsea B. Quenneville[1], Shira Tsour[1], Alexander O. Flynn-Carroll[1], Margaret M. Parker[1], Aimee M. Deaton[1], Patrick A. J. Haslett[1], Luca A. Lotta[2], Niek Verweij[2], Manuel A. R. Ferreira[2], Regeneron Genetics Center, Geisinger-Regeneron DiscovEHR Collaboration, Aris Baras[2], Gregory Hinkle[1], Paul Nioi[1]

1. Alnylam Pharmaceuticals, Cambridge, MA 02142
2. Regeneron Genetics Center, Tarrytown, NY 10591
*email: lward@alnylam.com

Supplementary Figures

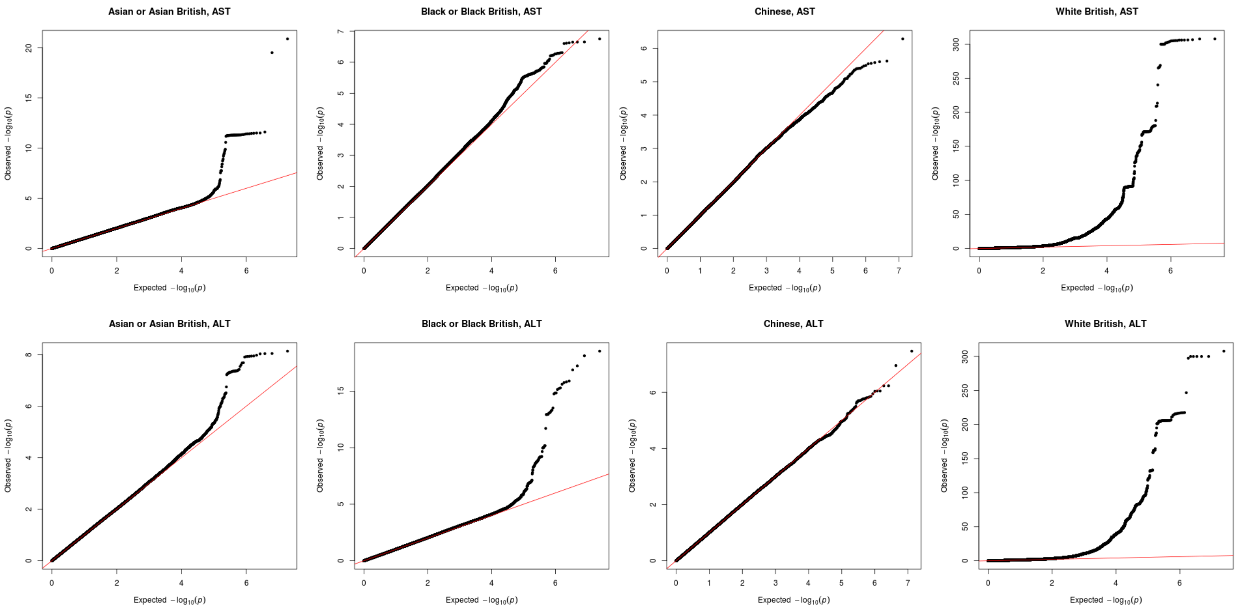

Supplementary Figure 1: Q-Q plots of GWAS p values (from SAIGE) for each sub-population and enzyme. Source data for this figure are available from NHGRI-EBI GWAS catalog accession GCST90013663 [https://www.ebi.ac.uk/gwas/studies/GCST90013663] and GCST90013664 [https://www.ebi.ac.uk/gwas/studies/GCST90013664].

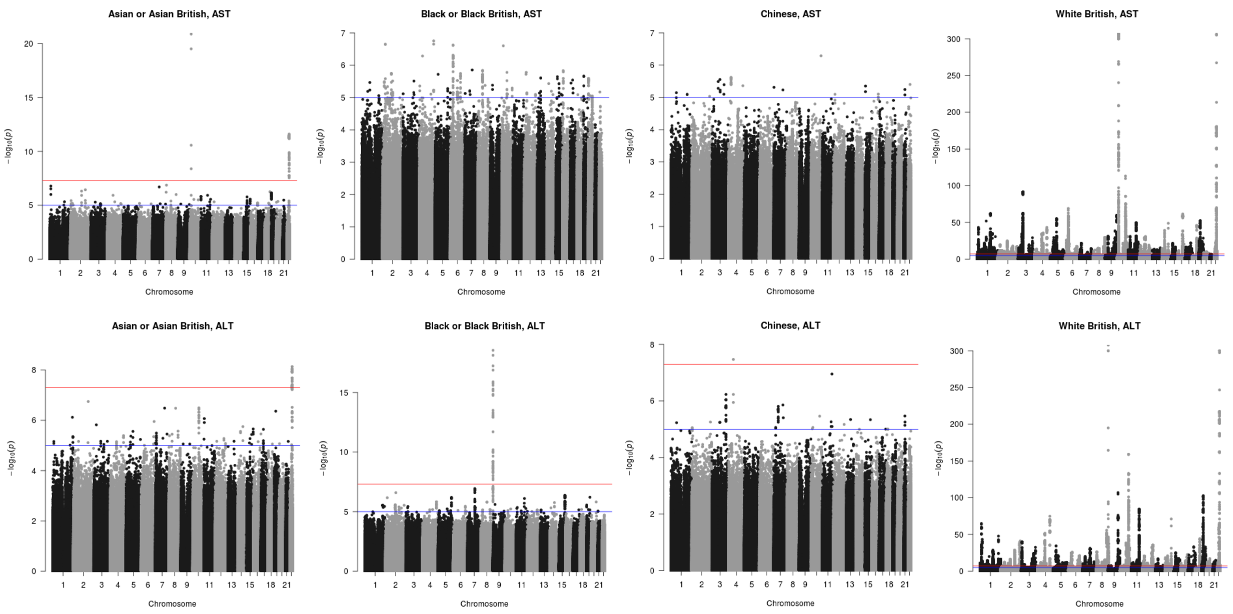

Supplementary Figure 2: Manhattan plots of GWAS p values (from SAIGE) for each sub-population and enzyme. Source data for this figure are available from NHGRI-EBI GWAS catalog accession GCST90013663 [https://www.ebi.ac.uk/gwas/studies/GCST90013663] and GCST90013664 [https://www.ebi.ac.uk/gwas/studies/GCST90013664].

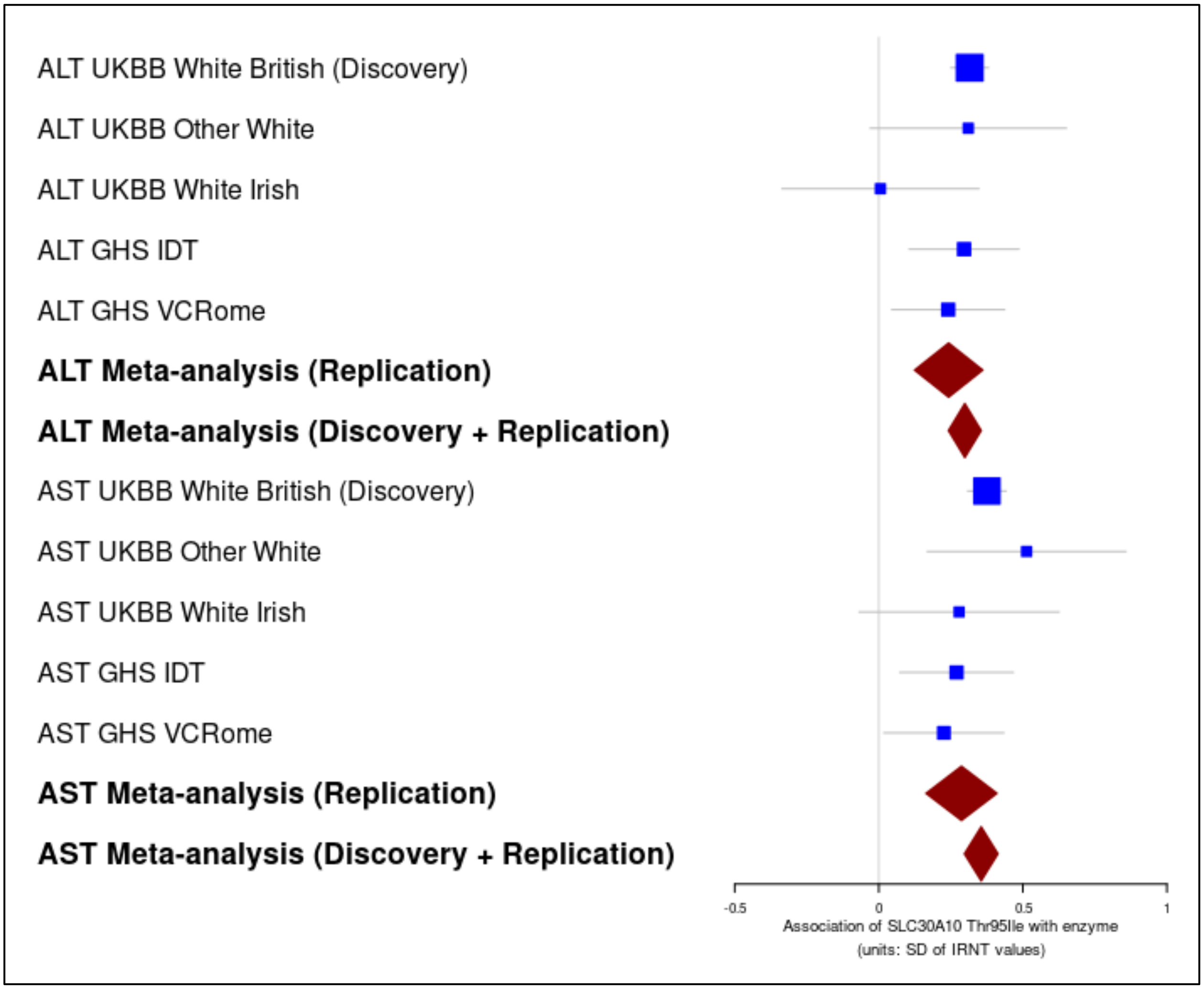

Supplementary Figure 3: Forest plot of *SLC30A10* Thr95Ile (rs188273166) association with ALT and AST in the discovery and replication groups. Boxes show effect size estimates from PLINK, and error bars show 95% confidence intervals. Source data in this figure are available in Supplementary Table 5.

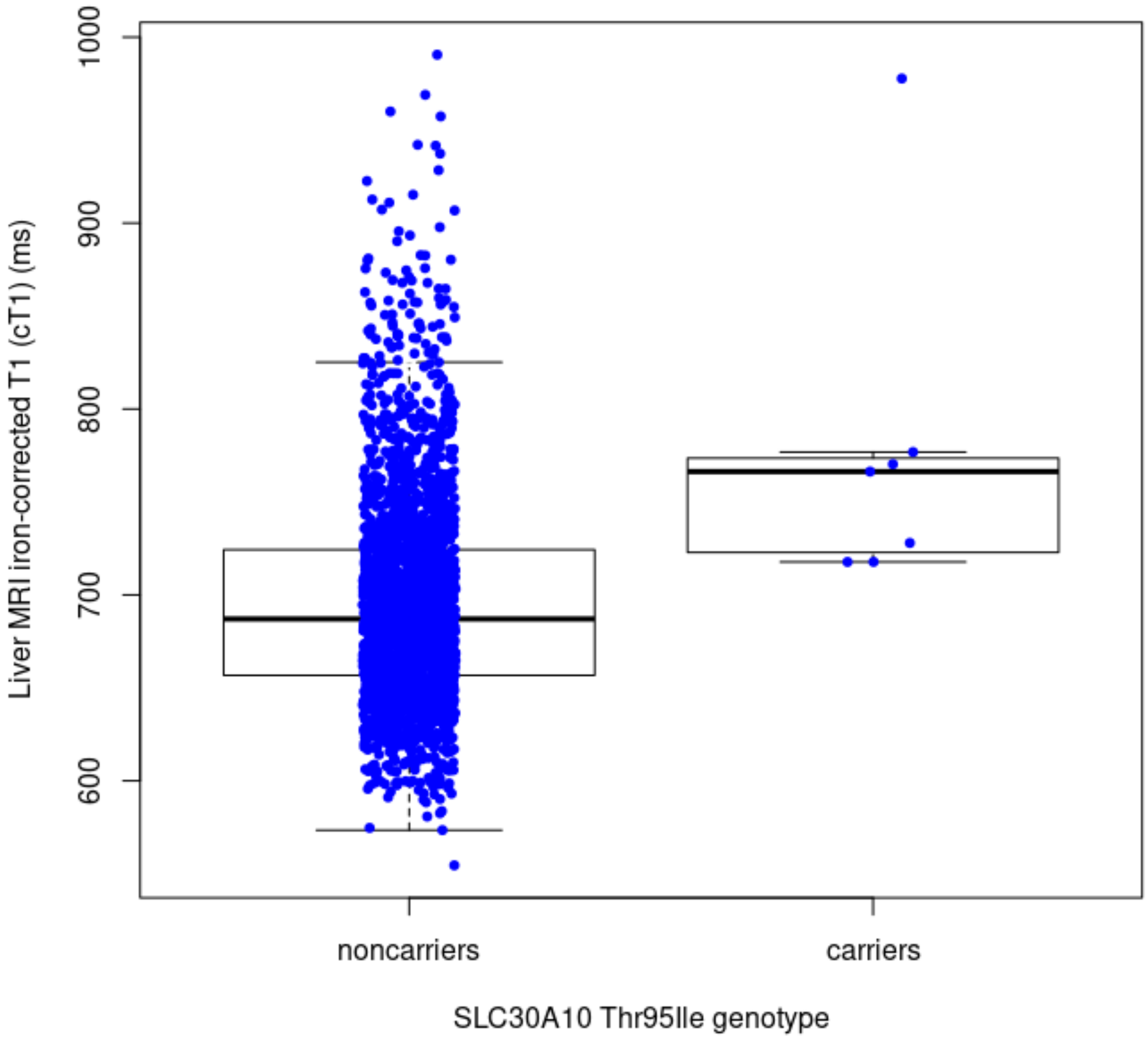

Supplementary Figure 4: iron-corrected T1 (cT1) values from liver MRI in seven SLC30A10 Thr95Ile carriers (limited to the White British population). Source data for this figure are available from the UK Biobank, Field 22417.

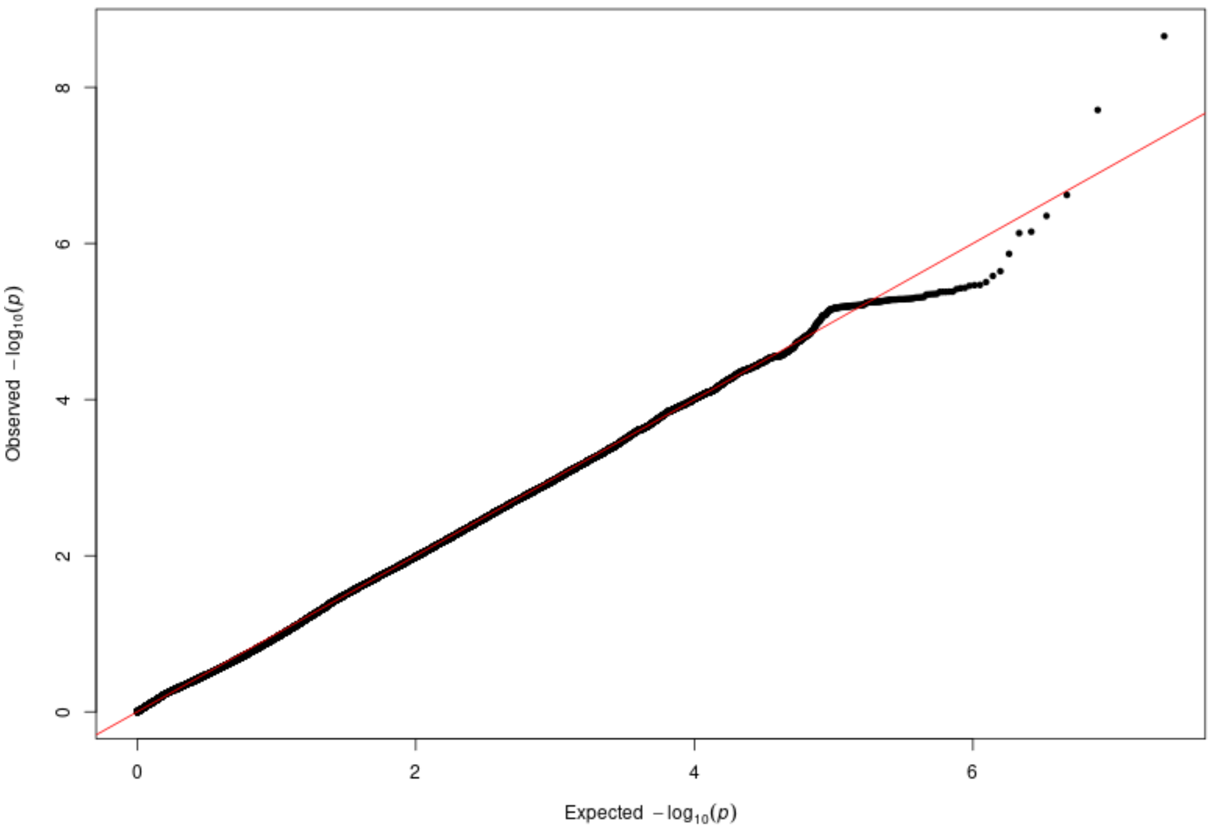

Supplementary Figure 5: Q-Q plot of GWAS (p value from SAIGE) for ICD10 diagnosis C24.0, extrahepatic bile duct cancer. Source data for this figure are available from NHGRI-EBI GWAS catalog accession GCST90013662 [https://www.ebi.ac.uk/gwas/studies/GCST90013662].

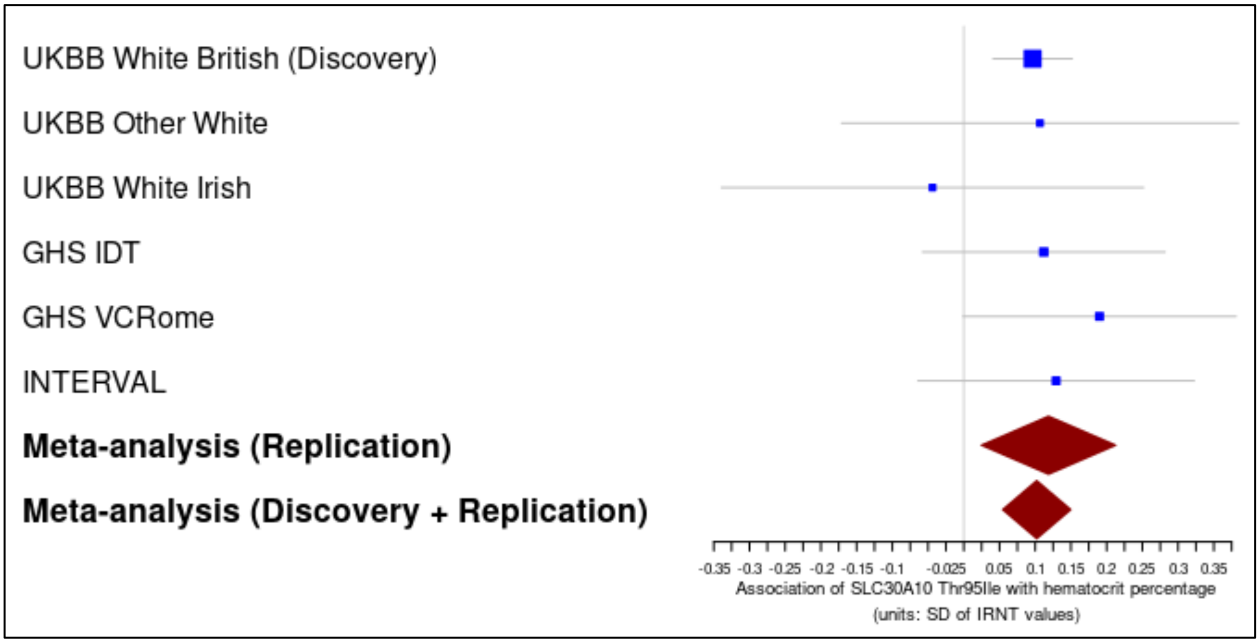

Supplementary Figure 6: Forest plot of *SLC30A10* Thr95Ile (rs188273166) association with hematocrit percentage in the discovery and replication groups. Boxes show effect size estimates from PLINK, and error bars show 95% confidence intervals. Source data in this figure are available in Supplementary Table 11.

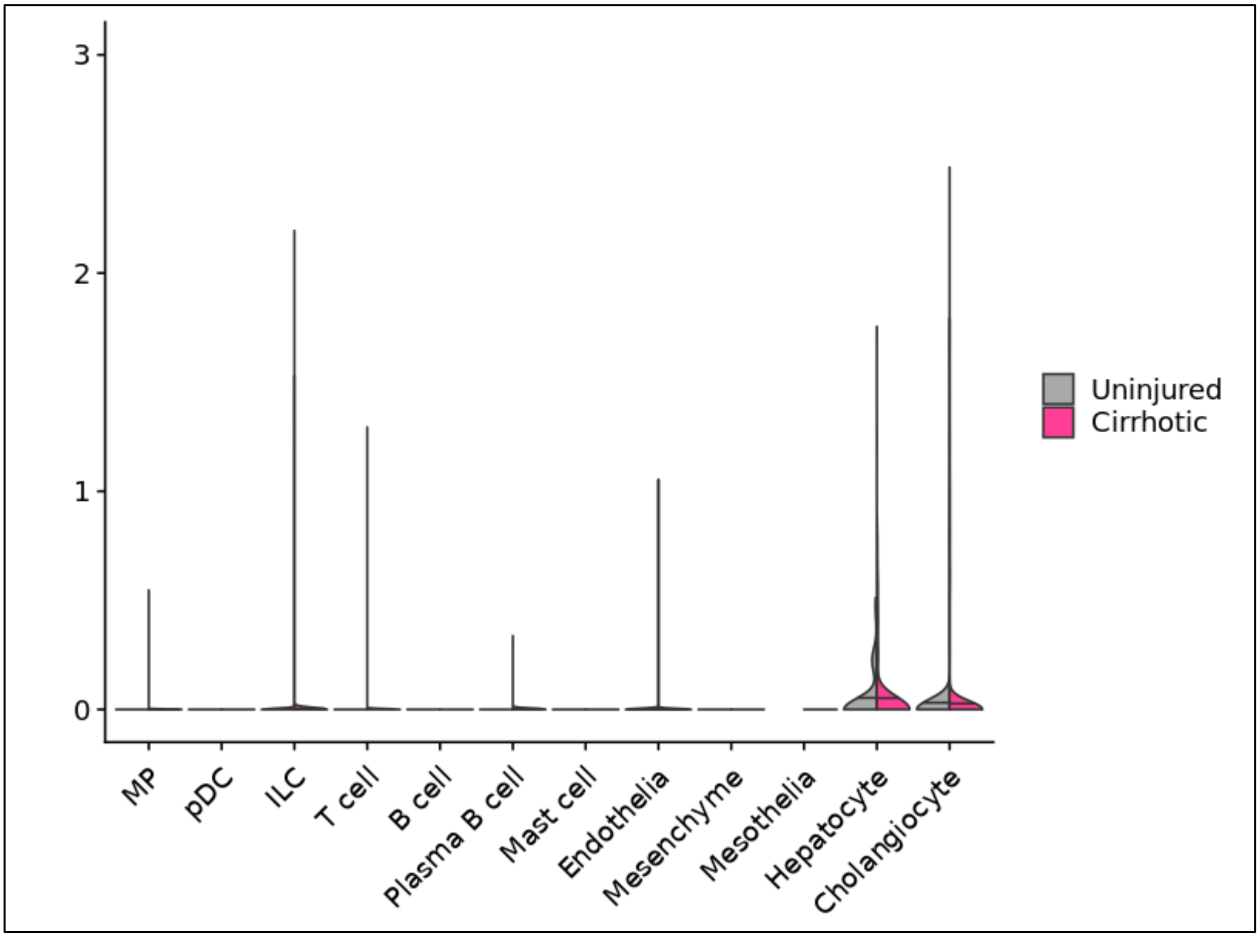

Supplementary Figure 7: Single-cell expression data, showing distribution of expression values for SLC30A10 from Ramachandran et al.[1] Produced by https://www.livercellatlas.mvm.ed.ac.uk/.

```
        10         20         30         40         50
MGRYSGKTCR LLFMLVLTVA FFVAELVSGY LGNSIALLSD SFNMLSDLIS
        60         70         80         90        100
LCVGLSAGYI ARRPTRGFSA TYGYARAEVV GALSNAVFLT ALCFTIFVEA
       110        120        130        140        150
VLRLARPERI DDPELVLIVG VLGLLVNVVG LLIFQDCAAW FACCLRGRSR
       160        170        180        190        200
RLQQRQQLAE GCVPGAFGGP QGAEDPRRAA DPTAPGSDSA VTLRGTSVER
       210        220        230        240        250
KREKGATVFA NVAGDSFNTQ NEPEDMMKKE KKSEALNIRG VLLHVMGDAL
       260        270        280        290        300
GSVVVVITAI IFYVLPLKSE DPCNWQCYID PSLTVLMVII ILSSAFPLIK
       310        320        330        340        350
ETAAILLQMV PKGVNMEELM SKLSAVPGIS SVHEVHIWEL VSGKIIATLH
       360        370        380        390        400
IKYPKDRGYQ DASTKIREIF HHAGIHNVTI QFENVDLKEP LEQKDLLLLC
       410        420        430        440        450
NSPCISKGCA KQLCCPPGAL PLAHVNGCAE HNGGPSLDTY GSDGLSRRDA
       460        470        480
REVAIEVSLD SCLSDHGQSL NKTQEDQCYV NRTHF
```

Supplementary Figure 8: Protein sequence of SLC30A10 along with experimental evidence cited by UNIPROT[2]. Underlined are the six transmembrane domains. Highlighted red is Thr95Ile. Highlighted in yellow are variants that demonstrated abolished Mn transport or membrane localization in vitro; highlighted in blue are variants that demonstrated lesser or no effect on Mn transport or membrane localization in vitro[3-5]. In bold are variants known to cause SLC30A10 deficiency (HMNDYT1)[5-7].

Supplementary Tables

	N	%
**Total individuals included in discovery GWAS**	411048	100.00%
**Individuals with ALT values available**	410949	99.98%
**Individuals with AST values available**	409627	99.65%
**Recruited in England**	363878	88.52%
**Recruited in Scotland**	29357	7.14%
**Recruited in Wales**	17813	4.33%
**Subpopulation: Asian or Asian British**	10894	2.65%
**Subpopulation: Black or Black British**	7594	1.85%
**Subpopulation: Chinese**	2356	0.57%
**Subpopulation: White or White British**	390204	94.93%
**Female**	222040	54.02%
**Self-reported diabetes at enrollment**	22118	5.38%
**Self-reported liver failure / cirrhosis at enrollment**	437	0.11%
**Self-reported hepatitis at enrollment**	2095	0.51%
**Current smoker**	41517	10.10%
**Former smoker**	140580	34.20%
**Never smoker**	227145	55.26%
**Current drinker**	378126	91.99%
**Former drinker**	14427	3.51%
**Never drinker**	17736	4.31%
	**mean**	**SD**
**Age at enrollment**	56.7	8.1
**BMI**	27.4	4.8
**Townsend deprivation index**	-1.4	3.0

Supplementary Table 1: demographic factors of participants in the discovery GWAS of ALT and AST

Enzyme	Population	Individuals tested (for SAIGE)	Variants tested	lambda GC
ALT	White British	387859	11811438	1.19
AST	White British	386570	11811438	1.21
ALT	Chinese	2338	6492165	0.99
AST	Chinese	2321	6492165	0.98
ALT	Black or Black British	7468	12049576	1.02
AST	Black or Black British	7426	12049576	1.02
ALT	Asian or Asian British	10635	9353299	1.02
AST	Asian or Asian British	10617	9353299	1.02

Supplementary Table 2: sample sizes, number of variants tested, and genomic inflation factors for each discovery GWAS

<table>
<tr><td colspan="2" rowspan="2"></td><th colspan="3">Exome genotype</th></tr>
<tr><th>Ref/Ref</th><th>Ref/Alt</th><th>Alt/Alt</th></tr>
<tr><th rowspan="3">Array genotype</th><th>Ref/Ref</th><td>300769</td><td>1</td><td>0</td></tr>
<tr><th>Ref/Alt</th><td>3</td><td>699</td><td>0</td></tr>
<tr><th>Alt/Alt</th><td>0</td><td>0</td><td>1</td></tr>
</table>

Supplementary Table 3: Comparison of array-based and exome-based genotype calls for SLC30A10 Thr95Ile

Ethnic group (self-identification)	*SLC30A10* Thr95Ile carriers	total array-typed	*SLC30A10* Thr95Ile carrier frequency	one carrier per
Do not know	1	204	0.49%	204
Prefer not to answer	2	1583	0.13%	792
White	3	544	0.55%	181
White British	1036	430230	0.24%	415
White Irish	35	12715	0.28%	363
Any other white background	30	15755	0.19%	525
Mixed	0	46	0.00%	
White and Black Caribbean	0	597	0.00%	
White and Black African	1	402	0.25%	402
White and Asian	3	802	0.37%	267
Any other mixed background	2	996	0.20%	498
Asian or Asian British	0	42	0.00%	
Indian	0	5660	0.00%	
Pakistani	1	1747	0.06%	1747
Bangladeshi	0	221	0.00%	
Any other Asian background	0	1747	0.00%	
Black or Black British	0	26	0.00%	
Caribbean	2	4297	0.05%	2149
African	0	3203	0.00%	
Any other Black background	0	118	0.00%	
Chinese	0	1508	0.00%	
Other ethnic group	1	4354	0.02%	4354
**Country of birth**				
Prefer not to answer	1	683	0.15%	683
England	860	379193	0.23%	441
Wales	45	21568	0.21%	479
Scotland	142	39132	0.36%	276
Northern Ireland	9	2990	0.30%	332
Republic of Ireland	12	4786	0.25%	399
Elsewhere	48	38297	0.13%	798
**Subpopulation for SAIGE analysis (with PCA outliers excluded)**				
White British	1054	408183	0.26%	387
White Irish	34	12259	0.28%	361
Other White	33	15767	0.21%	478
Asian or Asian British	5	11470	0.04%	2294
Black or Black British	2	8100	0.02%	4050

Supplementary Table 4: Self-reported ethnicity, country of birth, and subpopulation assignment for carriers of *SLC30A10* Thr95Ile

	Cohort	Carriers measured	Non-carriers measured	Total N	Method	Beta (units: SD of IRNT values)	SE	p
ALT	UKBB White British (Discovery)	790	320501	321291	PLINK	0.316	0.033	2.74E-21
ALT	UKBB Other White	31	13888	13919	PLINK	0.311	0.174	7.38E-02
ALT	UKBB White Irish	29	9693	9722	PLINK	0.006	0.174	9.74E-01
ALT	GHS IDT	90	57442	57532	BOLT	0.296	0.097	2.40E-03
ALT	GHS VCRome	78	51741	51819	BOLT	0.241	0.100	1.60E-02
**ALT**	**Meta-analysis (Replication)**	**228**	**132764**	**132992**	**METAL (classical)**	**0.242**	**0.061**	**6.46E-05**
**ALT**	**Meta-analysis (Discovery + Replication)**	**1018**	**453265**	**454283**	**METAL (classical)**	**0.299**	**0.029**	**1.51E-24**
AST	UKBB White British (Discovery)	788	319438	320226	PLINK	0.375	0.034	2.74E-28
AST	UKBB Other White	31	13834	13865	PLINK	0.513	0.176	3.57E-03
AST	UKBB White Irish	29	9661	9690	PLINK	0.279	0.177	1.16E-01
AST	GHS IDT	89	56855	56944	BOLT	0.270	0.101	7.30E-03
AST	GHS VCRome	75	51072	51147	BOLT	0.226	0.106	3.40E-02
**AST**	**Meta-analysis (Replication)**	**224**	**131422**	**131646**	**METAL (classical)**	**0.287**	**0.063**	**5.43E-06**
**AST**	**Meta-analysis (Discovery + Replication)**	**1012**	**450860**	**451872**	**METAL (classical)**	**0.355**	**0.030**	**1.74E-32**

Supplementary Table 5: Independent replication of ALT and AST associations with SLC30A10 Thr95Ile

Source	Study	Enzyme	Beta	SE	p	units	N	MAF
NHGRI-EBI GWAS Catalog: List of published studies with summary statistics (https://www.ebi.ac.uk/gwas/downloads/summary-statistics)	UK Household Longitudinal Study (Prins et al. 2017, PMID:28887542)	ALT	0.10	0.07	0.1454	log10 IU/L	5458	unknown
NHGRI-EBI GWAS Catalog: List of published studies with summary statistics (https://www.ebi.ac.uk/gwas/downloads/summary-statistics)	UK Household Longitudinal Study (Prins et al. 2017, PMID:28887542)	AST	0.14	0.09	0.1094	log units/L	5321	unknown
Accelerating Medicines Partnership - Common Metabolic Diseases Knowledge Portal (http://hugeamp.org/)	Exeter 10,000 Study (EXTEND)	ALT	0.65	unknown	0.1591	unknown	7159	unknown

Supplementary Table 6: Association of SLC30A10 Thr95Ile in available summary statistics from published ALT and AST GWAS

	Association with ALT (SAIGE p value in White British) when conditioning on:			
**Variant tested**	no other variants	*LYPLAL1_ZC3H11B* rs6541227	*SLC30A10* rs188273166	*MTARC1* rs2642438
LYPLAL1_ZC3H11B rs6541227	1.73E-16	NA	4.24E-16	3.39E-15
SLC30A10 rs188273166	1.55E-24	9.57E-25	NA	3.72E-25
MTARC1 rs2642438	1.83E-47	3.45E-46	4.84E-48	NA
	**Association with AST (SAIGE p value in White British) when conditioning on:**			
**Variant tested**	no other variants	*LYPLAL1_ZC3H11B* rs6541227	*SLC30A10* rs188273166	*MTARC1* rs2642438
LYPLAL1_ZC3H11B rs6541227	2.99E-09	NA	6.41E-09	7.95E-09
SLC30A10 rs188273166	4.93E-31	5.37E-31	NA	2.41E-31
MTARC1 rs2642438	9.10E+11	2.41E-10	4.62E-11	NA

Supplementary Table 7: Conditional association analysis demonstrating independence of ALT and AST associations at *SLC30A10*, *MTARC1*, and *LYPLAL1-ZC3H11B*

		rs188273166 genotype (Ref = major = G, Alt = minor = A) among white British unrelated subset	
		**G/G**	**G/A**
rs1776029 genotype (Ref = minor = A, Alt = major = G) among White British unrelated subset	**A/A**	12262	160
	**A/G**	102408	659
	**G/G**	216380	10
		**Het major**	**Carries minor**
	**Het major**	114670	819
	**Carries minor**	216380	10

Supplementary Table 8: Linkage between *SLC30A10* Thr95Ile (rs188273166) and blood manganese-associated SNP rs1776029

**quantitative_trait**	**SAIGE Pvalue**	**SAIGE beta**	**SAIGE se**	**SAIGE nobs**	**PLINK Pvalue**	**PLINK beta**	**PLINK se**	**PLINK nobs**
alanine_aminotransferase	2.87E-25	0.33	0.03	388903	1.36E-20	0.31	0.03	321184
albumin	3.93E-08	-0.19	0.03	356226	4.15E-06	-0.17	0.04	294253
apolipoprotein_a	2.64E-10	-0.20	0.03	354101	4.57E-08	-0.19	0.03	292509
aspartate_aminotransferase	1.54E-32	0.38	0.03	387611	5.26E-28	0.37	0.03	320120
hdl_cholesterol	1.77E-15	-0.25	0.03	356066	1.27E-10	-0.22	0.03	294127
haematocrit_pctge	2.14E-05	0.11	0.03	395899	6.60E-04	0.10	0.03	327042
haemoglobin_conc	1.15E-05	0.11	0.03	395899	5.92E-04	0.09	0.03	327041
gamma_glutamyltransferase	8.66E-05	0.12	0.03	388837	3.88E-04	0.12	0.03	321150
heel_bmd	2.29E-03	0.12	0.04	236955	2.17E-03	0.13	0.04	194196
corrected_t1	3.11E-03	1.20	0.41	2406	1.26E-03	1.22	0.38	1980

Supplementary Table 9: Selected association results of *SLC30A10* Thr95Ile with quantitative phenotypes after excluding participants with extrahepatic bile duct cancer

diagnosis	SAIGE pvalue	PLINK pvalue	PLINK beta	PLINK se	N_cases_white_british	N_carrier_cases_white_british	OR	OR 95% CI		pop_prevalence_white_british	expected	carrier_prevalence
D509_iron_deficiency_anaemia_unspecified	7.98E-04	0.00	0.55	0.15	14679	55	1.74	1.30	2.32	0.04	35.73	0.06
K830_cholangitis	2.26E-03	0.01	1.02	0.38	1239	9	2.76	1.31	5.84	0.00	3.02	0.01
D50_iron_deficiency_anaemia	3.16E-03	0.00	0.40	0.14	20060	69	1.49	1.14	1.95	0.05	48.82	0.07
K810_acute_cholecystitis	3.58E-03	0.00	0.95	0.29	2263	13	2.57	1.45	4.57	0.01	5.51	0.01
K831_obstruction_of_bile_duct	2.04E-02	0.03	0.81	0.38	1484	8	2.26	1.07	4.77	0.00	3.61	0.01
C221_intrahepatic_bile_duct_carcinoma	2.00E-01	0.14	1.05	0.71	336	2	2.86	0.71	11.52	0.00	0.82	0.00
C22_malignant_neoplasm_of_liver_and_intr	4.12E-01	0.25	0.66	0.58	755	3	1.94	0.62	6.05	0.00	1.84	0.00

Supplementary Table 10: Selected association results of *SLC30A10* Thr95Ile with quantitative phenotypes after excluding participants with extrahepatic bile duct cancer

Trait	Cohort	Carriers measured	Non-carriers measured	Total N	Method	Beta (units: SD of IRNT values)	SE	p
Hematocrit	UKBB White British (Discovery)	825	326329	327154	PLINK	0.096	0.0282443	6.44E-04
Hematocrit	UKBB Other White	32	14127	14159	PLINK	0.10667	0.141634	4.51E-01
Hematocrit	UKBB White Irish	30	9875	9905	PLINK	-0.044	0.150704	7.72E-01
Hematocrit	GHS IDT	96	61160	61256	BOLT	0.112	0.08657	2.00E-01
Hematocrit	GHS VCRome	81	54057	54138	BOLT	0.19	0.09751	5.10E-02
Hematocrit	INTERVAL	51	40470	40521	BOLT	0.129	9.86E-02	0.19
**Hematocrit**	**Meta-analysis (Replication)**	**290**	**179689**	**179979**	**METAL (classical)**	**0.1186**	**0.048**	**1.34E-02**
**Hematocrit**	**Meta-analysis (Discovery + Replication)**	**1115**	**506018**	**507133**	**METAL (classical)**	**0.102**	**0.024**	**2.72E-05**

Supplementary Table 11: Independent replication of hematocrit association with *SLC30A10* Thr95Ile

Study	Cluster	N samples	Mean expression (all)	N non-zero samples	Mean non-zero expression
MacParland et al.	Cholangiocytes	119	0.004103	1	0.488223
MacParland et al.	Hep 1 (Cluster 1)	1006	0.065215	66	0.994035
MacParland et al.	Hep 2 (Cluster 3)	909	0.08	76	0.956844
MacParland et al.	Hep 3 (Cluster 5)	629	0.033652	10	2.116684
MacParland et al.	Hep 4 (Cluster 6)	603	0.098785	32	1.861487
MacParland et al.	Hep 5 (Cluster 14)	202	0.099637	35	0.575048
MacParland et al.	Hep 6 (Cluster 15)	152	0.037035	2	2.814639
Aizarani et al.	Hepatocyte (Cluster 11)	1967	0.029491	54	1.074228
Aizarani et al.	Hepatocyte (Cluster 14)	683	0.026358	16	1.125168
Aizarani et al.	Hepatocyte (Cluster 17)	390	0.028209	10	1.100159
Aizarani et al.	Hepatocyte (Cluster 30)	46	0.043484	2	1.000122
Aizarani et al.	EPCAM+ cells and other cholangiocytes (Cluster 4)	599	0.023376	12	1.16685
Aizarani et al.	EPCAM+ cells and other cholangiocytes (Cluster 7)	379	0.005278	2	1.000122
Aizarani et al.	EPCAM+ cells and other cholangiocytes (Cluster...	27	0	0	NA
Aizarani et al.	EPCAM+ cells and other cholangiocytes (Cluster...	17	0	0	NA

Supplementary Table 12: Summary of *SLC30A10* single cell RNA-seq results from two liver expression atlases

Supplementary References

1 Ramachandran, P. *et al.* Resolving the fibrotic niche of human liver cirrhosis at single-cell level. *Nature* **575**, 512-518, doi:10.1038/s41586-019-1631-3 (2019).

2 Arnold, L. M., Hirsch, I., Sanders, P., Ellis, A. & Hughes, B. Safety and efficacy of esreboxetine in patients with fibromyalgia: a fourteen-week, randomized, double-blind, placebo-controlled, multicenter clinical trial. *Arthritis and rheumatism* **64**, 2387-2397, doi:10.1002/art.34390 (2012).

3 Zogzas, C. E., Aschner, M. & Mukhopadhyay, S. Structural Elements in the Transmembrane and Cytoplasmic Domains of the Metal Transporter SLC30A10 Are Required for Its Manganese Efflux Activity. *J Biol Chem* **291**, 15940-15957, doi:10.1074/jbc.M116.726935 (2016).

4 Zhao, Y., Feresin, R. G., Falcon-Perez, J. M. & Salazar, G. Differential Targeting of SLC30A10/ZnT10 Heterodimers to Endolysosomal Compartments Modulates EGF-Induced MEK/ERK1/2 Activity. *Traffic* **17**, 267-288, doi:10.1111/tra.12371 (2016).

5 Leyva-Illades, D. *et al.* SLC30A10 is a cell surface-localized manganese efflux transporter, and parkinsonism-causing mutations block its intracellular trafficking and efflux activity. *J Neurosci* **34**, 14079-14095, doi:10.1523/JNEUROSCI.2329-14.2014 (2014).

6 Quadri, M. *et al.* Mutations in SLC30A10 cause parkinsonism and dystonia with hypermanganesemia, polycythemia, and chronic liver disease. *American journal of human genetics* **90**, 467-477, doi:10.1016/j.ajhg.2012.01.017 (2012).

7 Tuschl, K. *et al.* Hepatic cirrhosis, dystonia, polycythaemia and hypermanganesaemia--a new metabolic disorder. *J Inherit Metab Dis* **31**, 151-163, doi:10.1007/s10545-008-0813-1 (2008).
