# Supplementary material for: GWAS of serum ALT and AST reveals an association of SLC30A10 Thr95Ile with hypermanganesemia symptoms
Source: Nat Commun. 2021 Jul 27;12:4571. doi: 10.1038/s41467-021-24563-1 (PMC8316433; doi:10.1038/s41467-021-24563-1)
Supplement: Supplementary file 2 — Descriptions of Additional Supplementary Files [file 41467_2021_24563_MOESM2_ESM.pdf]

Descriptions of Additional Supplementary Files

Supplementary Data 1
Description: GWAS results from SAIGE for each enzyme and population surpassing genome-wide significance threshold of p < 5 x 10-8.

Supplementary Data 2
Description: Annotation of 100 shared loci between ALT and AST GWAS, including PLINK effect size estimates with each enzyme and associations with liver disease.

Supplementary Data 3
Description: Sex-stratified analysis of effect sizes at the ALT and AST loci

Supplementary Data 4
Description: Summary of ALT and AST values, demographic characteristics, and drinking behavior in SLC30A10 Thr95Ile carriers vs. noncarriers.

Supplementary Data 5
Description: Linkage disequilibrium (|D'|) between SLC30A10 Thr95Ile and all nearby common GWAS catalog SNPs. Highlighted in bold are GWAS SNPs with |D'| > 0.9

Supplementary Data 6
Description: Phenome-wide association results of SLC30A10 Thr95Ile with quantitative phenotypes

Supplementary Data 7
Description: Phenome-wide association results of SLC30A10 Thr95Ile with ICD10 diagnosis codes
