# Supplementary material for: GWAS of serum ALT and AST reveals an association of SLC30A10 Thr95Ile with hypermanganesemia symptoms
Source: Nat Commun. 2021 Jul 27;12:4571. doi: 10.1038/s41467-021-24563-1 (PMC8316433; doi:10.1038/s41467-021-24563-1)
Supplement: Supplementary file 10 — Reporting Summary [file 41467_2021_24563_MOESM10_ESM.pdf]

nature research

Corresponding author(s): Lucas D. Ward

Last updated by author(s): Mar 17, 2021

# Reporting Summary

Nature Research wishes to improve the reproducibility of the work that we publish. This form provides structure for consistency and transparency in reporting. For further information on Nature Research policies, see our Editorial Policies and the Editorial Policy Checklist.

## Statistics

For all statistical analyses, confirm that the following items are present in the figure legend, table legend, main text, or Methods section.

| n/a | Confirmed | |
|---|---|---|
| ☐ | ☒ | The exact sample size (*n*) for each experimental group/condition, given as a discrete number and unit of measurement |
| ☐ | ☒ | A statement on whether measurements were taken from distinct samples or whether the same sample was measured repeatedly |
| ☐ | ☒ | The statistical test(s) used AND whether they are one- or two-sided<br>*Only common tests should be described solely by name; describe more complex techniques in the Methods section.* |
| ☐ | ☒ | A description of all covariates tested |
| ☐ | ☒ | A description of any assumptions or corrections, such as tests of normality and adjustment for multiple comparisons |
| ☐ | ☒ | A full description of the statistical parameters including central tendency (e.g. means) or other basic estimates (e.g. regression coefficient) AND variation (e.g. standard deviation) or associated estimates of uncertainty (e.g. confidence intervals) |
| ☐ | ☒ | For null hypothesis testing, the test statistic (e.g. *F*, *t*, *r*) with confidence intervals, effect sizes, degrees of freedom and *P* value noted<br>*Give P values as exact values whenever suitable.* |
| ☒ | ☐ | For Bayesian analysis, information on the choice of priors and Markov chain Monte Carlo settings |
| ☒ | ☐ | For hierarchical and complex designs, identification of the appropriate level for tests and full reporting of outcomes |
| ☐ | ☒ | Estimates of effect sizes (e.g. Cohen's *d*, Pearson's *r*), indicating how they were calculated |

*Our web collection on statistics for biologists contains articles on many of the points above.*

## Software and code

Policy information about availability of computer code

| | |
|---|---|
| Data collection | UK Biobank data were all collected by others, as described in previous publications, and were downloaded from the UK Biobank portal. Data were obtained under application number 26041. |
| Data analysis | All software used was publicly available. The following software was used for data analysis: R version 2.6.0 (base functions, and additional packages: aberrant, RNOmni v0.7.1, qqman v0.1.4, CMplot, forestplot); SAIGE; METAL, PLINK v1.90; BEDTools; ENSEMBL VEP. |

For manuscripts utilizing custom algorithms or software that are central to the research but not yet described in published literature, software must be made available to editors and reviewers. We strongly encourage code deposition in a community repository (e.g. GitHub). See the Nature Research guidelines for submitting code & software for further information.

## Data

Policy information about availability of data

All manuscripts must include a data availability statement. This statement should provide the following information, where applicable:

- Accession codes, unique identifiers, or web links for publicly available datasets
- A list of figures that have associated raw data
- A description of any restrictions on data availability

The source data underlying all figures are provided in the Supplementary Data file. Complete summary statistics from the genome-wide association studies of ALT, AST, and extrahepatic bile duct cancer have been submitted to the NHGRI-EBI GWAS catalog (https://www.ebi.ac.uk/gwas/).

# Field-specific reporting

Please select the one below that is the best fit for your research. If you are not sure, read the appropriate sections before making your selection.

- [x] Life sciences
- [ ] Behavioural & social sciences
- [ ] Ecological, evolutionary & environmental sciences

For a reference copy of the document with all sections, see nature.com/documents/nr-reporting-summary-flat.pdf

# Life sciences study design

All studies must disclose on these points even when the disclosure is negative.

**Sample size**: Sample size was not predetermined, and was instead based on the maximum sample size available in the UK Biobank dataset.

**Data exclusions**: The following subjects were excluded (this procedure was pre-determined): Individuals were chosen based on self-reported ethnicity (Field 21000) and ancestry outliers from each of the subgroups were excluded as described in the Methods.
Variants were excluded (this procedure was pre-determined): HWE p > 1e-12, missingness < 2% of samples, MAF > 0.1%, autosomes only.

**Replication**: Replication was successful in two other ancestry groups in the UK Biobank data and in two external cohorts from GHS-DiscovEHR.

**Randomization**: Association analysis was performed across all individuals. Covariates used were age, sex, BMI, and PC1-12 of genetic ancestry.

**Blinding**: Blinding was not relevant for the association analysis, and was not feasible for the cell staining analysis.

# Reporting for specific materials, systems and methods

We require information from authors about some types of materials, experimental systems and methods used in many studies. Here, indicate whether each material, system or method listed is relevant to your study. If you are not sure if a list item applies to your research, read the appropriate section before selecting a response.

## Materials & experimental systems

| n/a | Involved in the study | |
|---|---|---|
| ☐ | ☒ | Antibodies |
| ☐ | ☒ | Eukaryotic cell lines |
| ☒ | ☐ | Palaeontology and archaeology |
| ☒ | ☐ | Animals and other organisms |
| ☐ | ☒ | Human research participants |
| ☒ | ☐ | Clinical data |
| ☒ | ☐ | Dual use research of concern |

## Methods

| n/a | Involved in the study | |
|---|---|---|
| ☒ | ☐ | ChIP-seq |
| ☒ | ☐ | Flow cytometry |
| ☒ | ☐ | MRI-based neuroimaging |

## Antibodies

**Antibodies used**: Monoclonal ANTI-FLAG® M2-FITC, Clone M2 (dilution 1:100; Sigma-Aldrich, St. Louis, MO)
Calnexin Monoclonal Antibody, Clone AF18 (dilution 1:100; Invitrogen, Carlsbad, CA)

**Validation**: Validation statements are available on the manufacturers' websites.

## Eukaryotic cell lines

Policy information about cell lines

**Cell line source(s)**: HeLa cells (ATCC®, Manassas, VA)

**Authentication**: Cell line was not authenticated.

**Mycoplasma contamination**: Cell line was not tested for mycoplasma contamination.

**Commonly misidentified lines (See ICLAC register)**: Cell line is not commonly misidentified.

# Human research participants

Policy information about studies involving human research participants

| | |
|---|---|
| Population characteristics | Participants in UK Biobank have been described previously. Participants were healthy volunteers between 40 and 69 at enrollment. |
| Recruitment | Recruitment for UK Biobank, and the biases resulting, have been described by the UK Biobank (https://www.ukbiobank.ac.uk/2017/07/comparing-uk-biobank-participants-with-the-general-population/). Volunteers were more likely to be female and in better health than the general population. |
| Ethics oversight | Ethics oversight for the UK Biobank is provided by an Ethics and Governance Council. All research described was performed within the framework of Application 26041. |

Note that full information on the approval of the study protocol must also be provided in the manuscript.
